# Prevalence and risk factors of developmental language delay in a sample of children aged <6 years old in the Aseer region, Saudi Arabia: A community-based study

**DOI:** 10.1097/MD.0000000000043459

**Published:** 2025-07-25

**Authors:** Saleh M. Al-Qahtani, Hoda Ali Ahmed Shiba, Hebatalla Abdelmaksoud Abdelmonsef Ahmed, Ayed A. Shati, Layan Saeed Alshmrani, Razan Mubarak Alqahtani, Reema Saeed Almater, Rawan Alqahtani, Reema Mohammed Abdullah Alshehri, Yara Ahmed Salem Alshehri, Lamia Ahmed M. Saddah, Ramy Mohamed Ghazy

**Affiliations:** a Department of Child Health, College of Medicine, King Khalid University, Abha, Saudi Arabia; b Public Health and Community Medicine, Faculty of Medicine for Girls, Al-Azhar University, Cairo, Egypt; c Public Health and Community Medicine Department, Faculty of Medicine, Kafrelsheikh University, Kafr el-Sheikh, Egypt; d Department of Child Health, College of Medicine, King Khalid University, Abha, Saudi Arabia; e College of Medicine, King Khalid University, Abha, Saudi Arabia; f Tropical Health Department, High Institute of Public Health, Alexandria University, Alexandria, Egypt; g Community and Family Medicine Department, College of Medicine, King Khalid University, Abha, Saudi Arabia.

**Keywords:** child feeding, community engagement, mother education, preschool language scale, Saudi Arabia, speech delay

## Abstract

Universal screening for developmental language delay (DLD) is of vital importance, as early identification and intervention can significantly reduce the risk of long-term adverse outcomes, including diminished cognitive functioning, impaired communication skills, and literacy difficulties. This study aimed to determine the prevalence and risk factors associated with DLD in children. Using a community-based approach, a cross-sectional survey was conducted among residents of the Aseer region in Saudi Arabia. The study population included children aged ≤6 years. Face-to-face interviews were carried out in community settings, including nurseries and schools, where children were screened using the Arabic-translated version of the Preschool Language Scale, Fourth Edition. A total of 410 children were included; their median (interquartile range) age was 46.0 (22.0–65.0) months, 53.2% were females, 82.7% resided in urban areas, 38.5% of the studied parents reported a consanguineous relationship, and 8.5% of families reported a history of speech or language disorders. For auditory comprehension, infants exhibited the highest mean score, while kindergarten-aged children recorded the lowest mean score (105.1 ± 23.6 vs 80.7 ± 24.0; *P* < .001). Regarding expressive communication, infants had the highest performance, while preschoolers aged 37 to 48 months had the lowest score (101.2 ± 22.0 vs 79.4 ± 20.8; *P* < .001). Infants had the highest total score, while preschoolers had the lowest (113.7 ± 27.3 vs 80.9 ± 23.8; *P* < .001). The overall prevalence of DLD was 35.9% among all the studied children. Maternal education levels revealed a significant association, with a higher percentage of mothers in the DLD group having lower education levels compared with the non-DLD group (*P* = .035). A significant difference was related to the child’s nutrition, as 25.3% of children in the DLD group were breastfed compared with 45.4% who were bottle-fed (*P* = .001). DLD affects nearly one-third of the studied sample in the Aseer region, Saudi Arabia, and it was linked to maternal education and feeding practices. Implementaion of ommunity-based screening and intervention programs are necessary to reduce the burden of DLD.

## 1. Introduction

Speech entails the physical production of sounds, whereas language constitutes a broader spectrum encompassing comprehension, processing, and communication dynamics.^[1]^ Expressive language denotes proficiency in conveying meanings, predominantly through speech, yet extending to nonverbal forms such as gestures, signs, and written expressions. Conversely, receptive language pertains to comprehending others’ communications, involving auditory and visual skills.^[2]^

Speech and language disorders are prevalent in children with speech delay, known as developmental language delay (DLD), indicating a slower language development progression than their peers.^[3]^ Globally, the prevalence of speech delay among preschool children varies from 2.1% to 11.4%.^[4–6]^ The prevalence can reach up to 20% in the school-aged population.^[4,7,8]^ In healthy infants, the prevalence of DLD is 2 in 1000, but this rate is higher among high-risk infants, 60 in 1000.^[9]^ In Europe, around 5.8 million children and adolescents are affected by DLD.^[10]^

Across the Middle East and North Africa, Arabic is the native language, making it the fifth most widely spoken language globally.^[11,12]^ Despite the clear need for such studies, investigations into early language development in Arabic-speaking children are sparse, given the substantial typological differences in phonology, syntax, morphology, and orthography between Arabic and other languages like English. This absence restricts evidence-based approaches in educational and clinical settings, delaying the effective identification and treatment of early language disorders.^[13]^ A study in Saudi Arabia revealed that 45.5% of parents believed that their children had speech delays, with higher rates among 3- to 5-year olds.^[14]^ Children’s language difficulties can have harmful effects, leading to increased healthcare costs and financial burdens on families.^[15]^ Some children with DLD may have additional issues such as intellectual disabilities, autism spectrum disorders, or hearing loss, contributing to their language difficulties. In addition, children with DLD often face multiple challenges and misunderstandings that reinforce the stigma surrounding the condition.^[16]^ On the other hand, specific language impairment is diagnosed in children with primary language delay.^[6,17]^

The etiology of DLD can include a range of risk factors occurring at various stages of a child’s development.^[14,18,19]^ Prenatal factors include conditions such as preeclampsia, placental abruption, and intrauterine growth restriction, alongside maternal health issues such as multimorbidity and addiction.^[20]^ Additional risks arise from sociodemographic elements, including young maternal age, low educational attainment, multiple gestations, and single-parent households. Perinatally, preterm birth and cesarean delivery are important risk factors. Postnatally, factors such as inadequate breastfeeding, low birth weight, low Apgar score, and neonatal conditions such as cerebral hemorrhage and kernicterus further elevate the risk.^[21]^ In children, genetic predispositions, a family history of developmental communication, male gender, and low socioeconomic status also play critical roles. In addition, frequent otitis media can impair hearing and disrupt speech development, while certain suckling habits may lead to oro-motor dysfunction, compounding the challenges in language acquisition.^[14,18,19]^

Communities play a crucial role in health promotion and prevention by facilitating access to hard-to-reach groups, such as socially disadvantaged individuals and those with existing health conditions, without stigmatization.^[16,22]^ Children and adolescents, particularly those from disadvantaged backgrounds, are a key focus in primary prevention efforts, as they face greater health risks and fewer resources. By influencing living conditions across various settings, community-based interventions can help reduce social inequalities in children’s health and support their development and participation opportunities.^[23]^ This approach was proven to be successful in managing many health problems, including screening and management of diabetes^[24,25]^ and management of hypertension and other chronic diseases.^[26,27]^

Screening all children for DLD is crucial, as early diagnosis and intervention can mitigate long-term effects such as low intelligence quotient, communication challenges, and illiteracy. Moreover, it is essential to assess risk factors for developing effective prevention strategies and improving outcomes for children with speech and language disorders.^[14]^ For Arabic-speaking populations, existing assessments for DLD include the Modified Preschool Language Scale, Fourth Edition (PLS-4; Arabic version)^[28]^ and the Arabic Language Test.^[29]^ Despite screening recommendations and existing tools, surveys have repeatedly demonstrated that the majority of physicians do not perform routine screening using standardized tools.^[30]^ Despite the importance of screening, there is a significant gap in data regarding the prevalence of DLD and its associated risk factors in Saudi Arabia. Hence, this study aimed to determine the prevalence and risk factors associated with DLD in children aged 6 years or below.

## 2. Methods

### 2.1. Study design, setting, and participants

This cross-sectional survey was conducted among residents of the Aseer region in Saudi Arabia, encompassing urban and rural areas. The study population consisted of children aged ≤6 years. Children were categorized into infants (0–12 months), toddlers (13–24 months), early preschoolers (25–36 months), preschoolers (37–48 months), late preschoolers (49–60 months), and kindergarten-age children (61–72 months). Children from diverse socioeconomic backgrounds and geographic locations within the region were included. Through face-to-face interviews, we screened children in community settings such as nurseries and schools.

### 2.2. Sample size and sampling technique

The sample size was calculated using the Epi-Info software statistical package version 3.01. The criteria used for the calculation were as follows: 95% confidence level, 80% power of the study, and an expectation of a prevalence of DLD of 53.1%.^[14]^ Based on the previously mentioned criteria, the minimum required sample size was 383 participants. A total of 410 participants were surveyed between June 1 and July 31, 2024. Seven trained researchers recruited the required sample size using a non-random sampling design (convenience and snowball sampling techniques). The responses were successively recorded until the required sample size was reached.

### 2.3. Measurements and data collection tool

Parents who agreed to join the study were asked to complete a structured interviewer-based questionnaire.

The first section contained questions on the children’s and family’s sociodemographic characteristics, such as the child’s age, gender, residence, mother’s and father’s age at the child’s birth, mother’s and father’s education, mother’s and father’s occupation, family income, parental consanguinity, number of children in the family, child’s birth order, and family history of speech/language disorder.

The second section contained questions on pregnancy history, including prenatal, natal, and postnatal risk factors. Parents were asked whether the mother experienced any illnesses during pregnancy, the child’s birth conditions, the child’s postnatal health, the child’s nutrition and feeding practices, the child’s hearing and ear-related health, and whether the child had any deformities of the mouth or pharynx that could affect speech, such as cleft palate or other structural issues.

The third section contained questions on the parental assessment of speech development using a specific set of questions designed to compare the child’s speech abilities with peers from the parent’s perspective, such as the ability to express themselves, pronounce words clearly, converse easily with family members or friends, produce correct sentences, and speak Arabic like other children in Saudi Arabia and the parent’s satisfaction of the Arabic speaking of their children. Each question was scored from 0 to 3, where higher scores indicate better performance. The total score ranged from 0 to 18, with higher scores reflecting better speech development.^[31]^

The fourth section covers DLD assessment using the standardized, modified, Arabic-translated version of the PLS-4.^[28]^ The PLS-4 is an omnibus language test used to identify children with a language disorder. It is suitable for children between birth and the age of 6 and 11 months. Norms are reported in 2-month age bands for the first year and in 6-month age bands after that. It comprises auditory comprehension (assesses how much language the child understands through various age-appropriate tasks) and expressive communication (measures how well the child uses language to communicate) subscales through 62 and 68 tasks, respectively. Key areas include attention, play, vocal development, social communication, vocabulary, and preliteracy skills. Each subscale provides age-based standard scores, percentile ranks, and age equivalents. The total language score combines both subscales. To evaluate the child’s language abilities, raw scores are converted to standard scores using a table provided in the test manual. The standard scores are reported with a mean of 100 and a standard deviation of 15 relative to the child’s age group. A normal range is defined as a standard score between 77.5 and 122.5. Children scoring within this range are classified as “normal” (no-DLD). Conversely, children scoring <77.5 are definitively diagnosed with DLD. This standardized scoring system ensures consistency and reliability in the identification of DLD across different age groups. Internal consistency (Cronbach α) is high, particularly for total language scores (>0.90), ensuring reliable results.

### 2.4. Pilot study

Pilot testing of the study tool was conducted to assess the clarity of the questions, the time needed to complete the questionnaire, and the feasibility and response rate of participation before carrying out the actual study. Each of the 7 researchers responsible for data collection was asked to collect data from 5 to 10 participants to reach a total of 70 responses. After necessary modifications, all responses collected during the pilot study were omitted from the final analysis. The pilot study revealed that the response rate was 90%, and the questionnaire was comprehensible, needing 20 to 25 minutes to be completed.

### 2.5. Statistical analysis

The data were analyzed using the Statistical Package for the Social Sciences “SPSS version 22.0” software (IBM Corp., Armonk). Quantitative data normality was tested using the Kolmogorov-Smirnov test. Qualitative variables were described using numbers and percentages; the χ^2^ test was used for analysis, and the Monte Carlo exact test or the Fisher exact test was used if >20% of the expected cell value was <5. Numerical variables were expressed as means and standard deviations or medians (interquartile range [IQR]), and the Mann-Whitney *U* test and the Kruskal-Wallis test were used to compare groups. Spearman correlation analysis evaluated the relationship between the studied variables. Univariate logistic regression analysis was done to assess the effect of various study factors on the study’s outcomes, and the results were tabulated as odds ratios and 95% confidence intervals. A *P* value of <.05 was adopted as the level of significance.

## 3. Results

### 3.1. The general demographic characteristics of children and their families, with a comparison between children with and without DLD

Among the studied 410 children, the median (IQR) of the children’s age was 46.0 (22.0–65.0) months. The gender distribution of the participants revealed that there were slightly more females (53.2%) than males (46.8%). A majority of participants resided in urban areas (82.7%). The median age of mothers at the time of the child’s birth was 29.0 (25.0–34.0) years, while fathers had a median age of 33.0 (29.0–39.0) years. Educational attainment was high among both parents, with 69.0% of fathers and 62.7% of mothers having completed a university education. Regarding family income, 55.9% of participants reported an income between 5000 and 15,000 Saudi Arabian Riyal, with only (6.3%) earning <5000 Saudi Arabian Riyal. Approximately 38.5% of the studied parents reported a consanguineous relationship. The median number of children in families was 3.0 (2.0–4.0), with 12.2% of the studied children being the fourth child or beyond. Only 8.5% of families reported a history of speech or language disorders. Screen spending time data indicated that just over half of the children (48.3%) had >2 hours of screen time daily (Table 1).

**Table 1:** Comparison of child and family demographic characteristics regarding developmental language delay

Studied variables		Total (N = 410)	DLD (147 [35.9%])	No-DLD (263 [64.1%])	*P* value
Child’s age (months)	Median (IQR)	46.0 (22.0–65.0)	48.0 (33.0–61.0)	44.0 (13.0–69.0)	.167^U^
	0–12	71 (17.3%)	8 (11.3%)	63 (88.7%)	<.001*^X^
	13–24	60 (14.6%)	16 (26.7%)	44 (73.3%)	
	25–36	90 (22%)	32 (35.6%)	58 (64.4%)	
	37–48	72 (17.6%)	36 (50.0%)	36 (50.0%)	
	49–60	69 (16.8%)	33 (47.8%)	36 (52.2%)	
	61–72	48 (11.7%)	22 (45.8%)	26 (54.2%)	
Gender	Male	192 (46.8%)	66 (34.4%)	126 (65.6%)	.558^X^
	Female	218 (53.2%)	81 (37.2%)	137 (62.8%)	
Residence	Urban	339 (82.7%)	124 (36.6%)	215 (63.4%)	.504^X^
	Rural	71 (17.3%)	23 (32.4%)	48 (67.6%)	
Mother’s age at child’s birth (yr)	Median (IQR)	29.0 (25.0–34.0)	28.0 (25.0–34.0)	30.0 (25.0–34.0)	.306^U^
Father’s age at child’s birth (yr)	Median (IQR)	33.0 (29.0–39.0)	32.0 (29.0–39.0)	33.0 (29.0–39.0)	.547^U^
Father’s education	Illiterate	0 (0%)	0 (0.0%)	0 (0.0%)	.185^X^
	Primary/intermediate	17 (4.1%)	9 (52.9%)	8 (47.1%)	
	Secondary	82 (20%)	31 (37.8%)	51 (62.2%)	
	University	283 (69%)	101 (35.7%)	182 (64.3%)	
	Postgraduate	28 (6.8%)	6 (21.4%)	22 (78.6%)	
Mother’s education	Illiterate	6 (1.5%)	2 (33.3%)	4 (66.7%)	.035*^MC^
	Primary/intermediate	23 (5.6%)	9 (39.1%)	14 (60.9%)	
	Secondary	101 (24.6%)	49 (48.5%)	52 (51.5%)	
	University	257 (62.7%)	79 (30.7%)	178 (69.3%)	
	Postgraduate	23 (5.6%)	8 (34.8%)	15 (65.2%)	
Father’s occupation	Government employee	323 (78.8%)	122 (37.8%)	201 (62.2%)	.214^MC^
	Private business	74 (18%)	20 (27%)	54 (73%)	
	Unemployed	13 (3.2%)	5 (38.5%)	8 (61.5%)	
Mother’s occupation	Employed	150 (36.6%)	53 (35.3%)	97 (64.7%)	.867^X^
	Unemployed	260 (63.4%)	94 (36.2%)	166 (63.8%)	
Family income, SAR	<5000	26 (6.3%)	11 (42.3%)	15 (57.7%)	.159^X^
	5000–15,000	229 (55.9%)	81 (35.4%)	148 (64.6%)	
	**>**15,000–20,000	122 (29.8%)	38 (31.1%)	84 (68.9%)	
	**>**20,000	33 (8%)	17 (51.5%)	16 (48.5%)	
Parental relation (consanguinity)	Yes	158 (38.5%)	51 (32.3%)	107 (67.7%)	.232^X^
	No	252 (61.5%)	96 (38.1%)	156 (61.9%)	
Number of children in family	Median (IQR)	3.0 (2.0–4.0)	3.0 (2.0–4.0)	3.0 (2.0–4.0)	.919^U^
Child’s birth order	First to second	245 (59.8%)	97 (39.6%)	148 (60.4%)	.782^X^
	Third to fourth	115 (28%)	37 (32.2%)	78 (67.8%)	
	> Fourth	50 (12.2%)	13 (26%)	37 (74%)	
Family history of speech/language disorder	Yes	35 (8.5%)	16 (45.7%)	19 (54.3%)	.203^X^
	No	375 (91.5%)	131 (34.9%)	244 (65.1%)	
How much screen time does the child have (TV, mobile phone, or laptop)?	<2 h	212 (51.7%)	81 (38.2%)	131 (61.8%)	.304^X^
	≥2 h	198 (48.3%)	66 (33.3%)	132 (66.7%)	

DLD = developmental language delay, IQR = interquartile range, MC = Monte Carlo test, SAR = Saudi Arabian Riyal (1 USD = 3.75 SAR), U = Mann-Whitney *U* test, X = χ^2^ test.

* Significant.

The overall prevalence of DLD was 35.9% among all the studied children. Moreover, the results indicate a significant association between age groups and DLD (*P* < .001). In the 0 to 12 childrens’ age group, 11.3% showed delays. In the toddler category (13–24 months), 26.7% had DLD. The highest prevalence of DLD was noted in the preschooler group (37–48 months), where half of the children (50.0%) showed DLD. Maternal education levels revealed a significant association, with a higher percentage of mothers in the DLD group having lower education levels than the non-DLD group (*P* = .035). Other demographic factors, such as sex, residence, parental ages at childbirth, father’s occupation, family income, family history, and parenteral consanguinity, did not show significant differences (*P* > .05; Table 1).

### 3.2. The perinatal and postnatal health history of the study parents, with a comparison between children with and without DLD

A small percentage of mothers (10.2%) reported experiencing illnesses during pregnancy, with the majority (89.8%) reporting no health complications. Regarding the delivery process, 13.9% of mothers had a difficult delivery experience. In addition, 10.7% of the deliveries were preterm (before 37 weeks of gestation). The data on birth weight showed that 14.6% of children weighed <2500 grams at birth. Postnatal complications were present among 19.0% of the studied sample. Nutritional practices revealed that more than half (52.7%) were bottle-fed. Almost half of the children (48.5%) used pacifiers. Most children did not experience any hearing problems (98.0%). However, 11.7% had experienced a middle ear infection, and 17.6% had other ear-, nose-, or throat-related illnesses. Only 0.5% of children reported having deformities in the mouth or pharynx.

Regarding the comparison between children with DLD and those without DLD, a significant difference was related to the child’s nutrition, as 25.3% of children in the DLD group were breastfed compared with 45.4% in the group without DLD (*P* = .001). Other factors, including maternal illnesses during pregnancy, difficult delivery, prematurity, low birth weight, and postnatal health problems, did not differ significantly between the studied groups (*P* > .05; Table 2).

**Table 2:** Comparison of perinatal and postnatal health history regarding developmental language delay

Studied variables		Total (N = 410)	DLD (147 [35.9%])	No-DLD (263 [64.1%])	*P* value
Did the mother suffer from any illnesses during pregnancy?	Yes	42 (10.2%)	12 (28.6%)	30 (71.4%)	.299^X^
	No	368 (89.8%)	135 (36.7%)	233 (63.3%)	
Was the delivery difficult?	Yes	57 (13.9%)	15 (26.3%)	42 (73.7%)	.106^X^
	No	353 (86.1%)	132 (37.4%)	221 (62.6%)	
Was the delivery premature (before 37 week of gestation)?	Yes	44 (10.7%)	13 (29.5%)	31 (70.5%)	.356^X^
	No	366 (89.3%)	134 (36.6%)	232 (63.4%)	
Was the child’s weight <2500 g at birth?	Yes	60 (14.6%)	16 (26.7%)	44 (73.3%)	.108^X^
	No	350 (85.4%)	131 (37.4%)	219 (62.6%)	
Did the child experience any health problems after birth?*	Yes	78 (19.0%)	21 (26.9%)	57 (73.1%)	.068^X^
	No	332 (80.9%)	126 (38%)	206 (62%)	
How was the child’s nutrition?	Breastfeeding	194 (47.3%)	49 (25.3%)	145 (74.7%)	.001†^X^
	Bottle feeding	216 (52.7%)	98 (45.4%)	118 (54.6%)	
Did the child use of pacifiers?	Yes	199 (48.5%)	70 (35.2%)	129 (64.8%)	.781^X^
	No	211 (51.5%)	77 (36.5%)	134 (63.5%)	
Is there any problem with hearing?	Yes	8 (2.0%)	4 (50.0%)	4 (50.0%)	.465^FE^
	No	402 (98.0%)	143 (35.6%)	259 (64.4%)	
Has the child ever had ottitis media?	Yes	48 (11.7%)	14 (29.2%)	34 (70.8%)	.304^X^
	No	362 (88.3%)	133 (36.7%)	229 (63.3%)	
Has the child ever had any other ear, nose, or throat-related illnesses?	Yes	72 (17.6%)	20 (27.8%)	52 (72.2%)	.116^X^
	No	338 (82.4%)	127 (37.6%)	211 (62.4%)	
Does the child have any deformity in the mouth or pharynx?	Yes	2 (0.5%)	1 (50.0%)	1 (50.0%)	<.999^FE^
	No	408 (99.5%)	146 (35.8%)	262 (64.2%)	

DLD = developmental language delay, FE = Fisher exact test, X = χ^2^ test.

* Cyanosis, jaundice, convulsions, meningitis, or admission to Intensive Care Unit for more than 2 days.

† Significant.

### 3.3. Parental assessment of speech development

**Figure 1.**
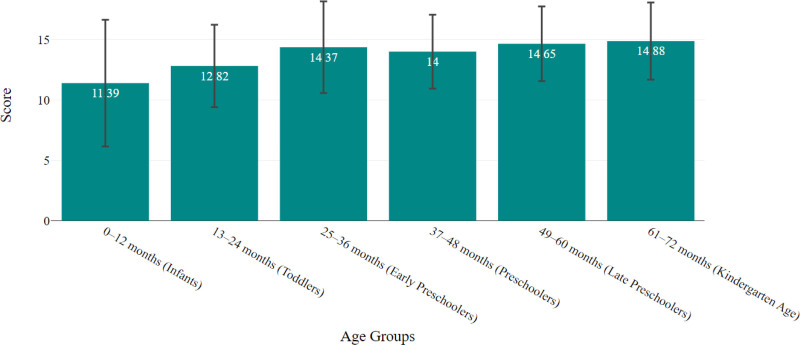
Parental assessment of speech development mean scores across age groups. Error bars represent standard deviations from the mean score. Infants (0–12 mo) have the lowest mean score of 11.4, while the lowest mean score of toddlers (13–24 mo) is 12.8. Early preschoolers (25–36 mo) show a notable increase to 14.4. The scores of preschoolers (37–48 and 49–60 mo) are 14.0 and 14.6, respectively, and the kindergarten group (61–72 mo) achieves the highest score of 14.9.

The infants (0–12 months) exhibited the lowest mean score of 11.4. As children transition to the toddler stage (13–24 months), the mean score rises slightly to 12.8. The early preschoolers (25–36 months) show a marked increase in the mean score to 14.4. The scores for the preschool age groups (37–48 and 49–60 months) were 14.0 and 14.6, respectively. Finally, the kindergarten-age group (61–72 months) has the highest mean score of 14.9 (Fig. 1).

### 3.4. DLD screening using the Arabic version of PLS-4

Table 3 compares the PLS-4 standardized scores across different age groups, including auditory comprehension, expressive communication, and total scores. For auditory comprehension, infants aged 0 to 12 months exhibited the highest mean score (105.1 ± 23.6). In contrast, kindergarten-aged children (61–72 months) recorded the lowest mean score (80.7 ± 24.0), with statistically significant differences (*P* < .001). Regarding expressive communication, the infant age group showed the highest performance, with a mean score of 101.2 ± 22.0, while preschoolers aged 37 to 48 months had the lowest score (79.4 ± 20.8), with statistically significant differences (*P* < .001). The total scores, combining auditory comprehension and expressive communication, showed that the infants had the highest total score (113.7 ± 27.3); scores declined through the early preschool years, reaching the lowest mean score of 80.9 ± 23.8 in preschoolers aged 37 to 48 months, with statistically significant differences (*P* < .001).

**Table 3:** Age group comparison of PLS-4 standardized scores

Age groups (mo)	PLS-4		
	Auditory comprehension, mean ± SD	Expressive communication, mean ± SD	Total score, mean ± SD
0–12 (infants)	105.1 ± 23.6	101.2 ± 22.0	113.7 ± 27.3
13–24 (toddlers)	100.5 ± 21.4	92.2 ± 25.9	94.2 ± 27.5
25–36 (early preschoolers)	91.1 ± 19.9	88.2 ± 25.5	88.7 ± 25.7
37–48 (preschoolers)	84.1 ± 24.4	79.4 ± 20.8	80.9 ± 23.8
49–60 (late preschoolers)	91.5 ± 30.3	88.5 ± 26.3	89.2 ± 29.1
61–72 (kindergarten age)	80.7 ± 24.0	88.7 ± 23.0	83.5 ± 25.6
*P* value	<.001*^W^	<.001*^W^	<.001*^W^

PLS-4 = Preschool Language Scale, Fourth Edition, SD = standard deviation, W = Kruskal-Wallis test.

* Significant.

The post hoc pairwise comparisons of PLS-4 standardized scores across different age groups revealed significant differences in auditory comprehension, expressive communication, and total scores. Infants (0–12 months) exhibited significantly higher scores than early preschoolers (25–36 months), preschoolers (37–48 months), late preschoolers (49–60 months), and kindergarten-age children (61–72 months) across all domains, indicating a decline in skills with age. Toddlers (13–24 months) also showed significantly higher scores compared with preschoolers and kindergarten-age children (Table S1, Supplemental Digital Content, https://links.lww.com/MD/P445).

**Figure 2.**
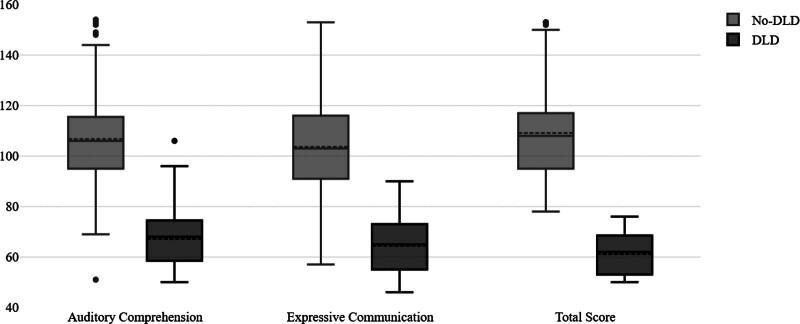
Comparison of developmental language delay (DLD) groups using Preschool Language Scale, Fourth Edition (PLS-4) standardized scores. Box plot comparison of PLS-4 standardized scores between children with DLD and those without DLD across auditory comprehension, expressive communication, and total score. The DLD group shows lower scores in all domains.

Figure 2 compares receptive language, expressive language, and total standardized scores between children with and without DLD. For receptive language scores, children with no-DLD demonstrated a higher median score of 106.0 (IQR, 95.0–116.0). In contrast, children with DLD had a lower median score of 68.0 (IQR, 58.0–75.0). Similarly, expressive language scores were markedly better in the no-DLD group, with a median of 103.0 (IQR, 91.0–116.0). The DLD group showed a lower median of 65.0 (percentile range, 55.0–73.0). For the total scores, the children with no-DLD achieved the highest scores, with a median of 108.0 (IQR, 95.0–117.0). Conversely, the DLD group had a median of 62.0 (IQR, 53.0–69.0).

Univariate logistic regression analysis of DLD according to child and family demographic characteristics revealed non statistically significant associations between most demographic characteristics and DLD. However, secondary education of the mother was associated with higher odds of DLD in children (odds ratio [OR], 2.1 [95% CI, 1.3–3.4]; *P* = .002), and higher birth order was associated with reduced odds of DLD (OR, 0.5 [95% CI, 0.3–0.8]; *P* = .004; Table S2, Supplemental Digital Content, https://links.lww.com/MD/P446).

The univariate logistic regression analysis of DLD according to perinatal and postnatal health history indicated that breastfeeding was significantly associated with lower odds of DLD compared with bottle feeding (OR, 0.4 [95% CI, 0.3–0.6]; *P* < .001). Other variables, such as maternal illness during pregnancy, delivery complications, prematurity, low birth weight, postnatal health issues, pacifier use, hearing problems, ear infections, and oral deformities, did not show significant associations with DLD (Table S3, Supplemental Digital Content, https://links.lww.com/MD/P447).

### 3.5. Relation between parental assessment scores of speech delay and PLS-4 standardized total scores

**Figure 3.**
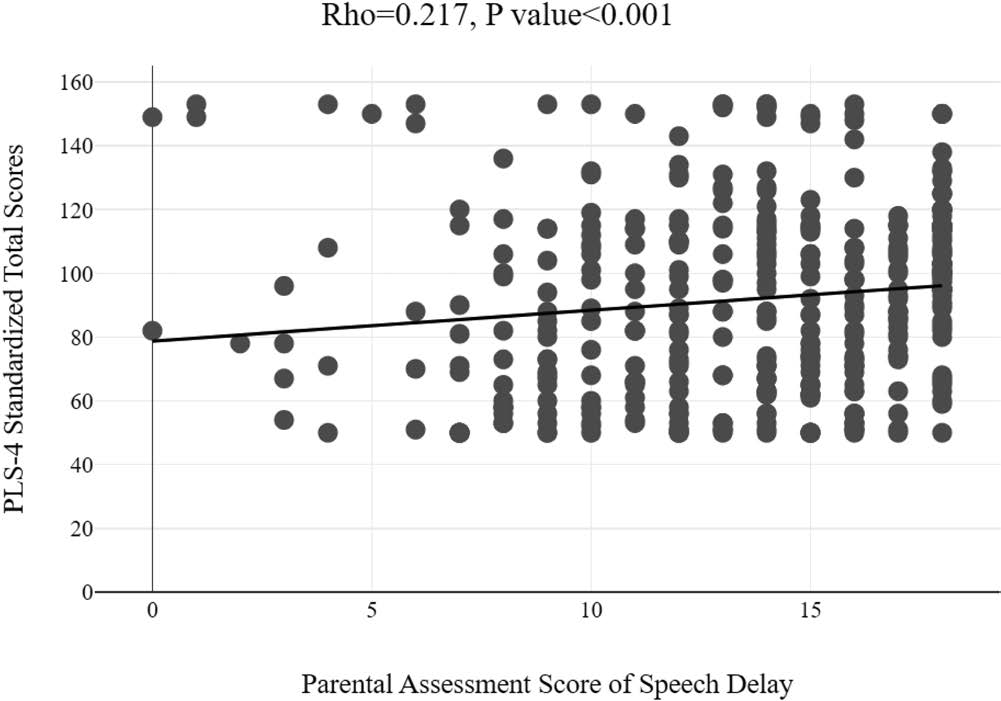
Correlation of parental assessment scores with Preschool Language Scale, Fourth Edition (PLS-4) standardized scores. A statistically significant positive correlation between parental assessment scores and PLS-4 standardized speech development scores.

Figure 3 illustrates the correlation between parental assessment scores of speech delay and PLS-4 standardized total scores. A statistically significant positive correlation was observed (ρ = 0.217; *P* < .001).

## 4. Discussion

Screening for developmental delays and their determinants early in life is crucial for implementing effective interventions promoting healthy development and well-being. This study found a prevalence of DLD of 35.9% among children under the age of 7 years according to PLS-4. Other research from various countries reported a wide range of DLD prevalence rates from 2.1% to 20%.^[4–8]^ Such variability is often attributed to differences in screening tools and the age groups being assessed. In Saudi Arabia, there are limited data regarding the prevalence of DLD. However, the notably high prevalence of DLD among Saudi children was explained by Alzahrani et al^[14]^ to be linked to several factors, including the early introduction of complementary foods before 6 months of age, closely spaced births, and lower levels of maternal education within the studied population. These factors can significantly impact a child’s development, highlighting the need for targeted public health initiatives and educational programs aimed at parents.

Age-disaggregated data revealed a significant association between age and DLD. Infants (0–12 months) showed the lowest speech delay prevalence (11.3%). However, in the preschoolers’ category (37–48 months), 50.0% showed DLD. In line with the findings of Metwally et al,^[32]^ children aged 3 to 6 years are significantly more likely to be diagnosed with DD compared with younger age groups. A recent study conducted in Saudi Arabia on children aged 16 to 36 months found that biological factors and excessive screen time were significant contributors to DLD in this age group.^[33]^ Moreover, this increased prevalence in the 3- to 6-year age range can be attributed to other risk factors, including familial violence, a lack of stimulating environments, chronic malnutrition, and decreased immunity, which can lead to heightened vulnerability to diseases. These issues may stem from inadequate weaning practices or unhealthy dietary behaviors, which are critical during this developmental stage.^[34,35]^

Disaggregating DLD by sex revealed that females exhibited a relatively higher prevalence of DLD (37.2%) compared with males (34.4%). On the contrary, a strong male sex association with DLD has been consistently reported, suggesting that biological, social, or environmental factors may contribute to this disparity.^[32,36]^ The contradictory findings in this study may indicate variations in sample characteristics, diagnostic criteria, or cultural influences on language development. Moreover, the absence of statistically significant associations may, in part, be attributed to the nonrandom sampling approach, which may have influenced the distribution of key risk factors within the study population.

The assessment of speech development using a parental questionnaire revealed an increasing pattern in mean scores across different age groups with infants aged 0 to 12 months having the lowest mean score of 11.4 and kindergarten children (61–72 months) achieving the highest mean score of 14.9, reflecting their advanced speech development as they prepare for more formal education. It was previously shown that speech progression reflects the typical trajectory of language development, where foundational speech skills begin to emerge in infancy and evolve into more complex language abilities as children approach school age.^[37]^ These results underscore the importance of early childhood experiences and interactions in fostering language skills.

This study identified low maternal education to be associated with DLD. This finding aligns with the research conducted by Metwally et al,^[32]^ which revealed that low social class, including low maternal education, is a significant predictor for delays across all developmental language domains in Egyptian children. Families facing socioeconomic disadvantages often encounter numerous challenges related to basic services, including employment, housing, food security, and healthcare.^[38]^ Moreover, children whose parents have attained a college education or higher are less likely to experience any type of developmental delay. Lower educational levels among parents tend to correlate with decreased cognitive stimulation and responsiveness to cultural values, which can inhibit access to health education and limit participation in enriching learning activities designed to enhance children’s mental development. In addition, parents with lower educational backgrounds may have restricted access to the internet and social media, further limiting their resources for supporting their children’s growth and development.^[39,40]^

Breast milk plays a crucial role in the development of children’s language skills, as it contains essential unsaturated fats that are easily absorbed by infants and beneficial for their metabolism and overall growth.^[41]^ In this study, approximately one-quarter (25.4%) of breastfed children had DLD compared with nearly half (45.4%) of those who were bottle-fed. This disparity underscores the potential impact of breastfeeding on language development. Supporting this observation, a meta-analysis by Abida et al^[42]^ concluded that breastfeeding positively influences children’s language acquisition, highlighting the importance of promoting breastfeeding as a key factor in fostering healthy communication skills in early childhood.

In addition, this study found birth order to be associated with DLD where higher birth order was associated with reduced odds of DLD. On the contrary, traditional evidence suggests that first-born children typically demonstrate superior linguistic outcomes compared with their later-born siblings. This advantage is often attributed to the increased parental attention and more frequent verbal interactions that first-born children receive during their early developmental years.^[43]^ Zajonc and Markus^[44]^ introduced the “confluence model,” which posits that the intellectual environment within a family diminishes as the number of children increases, potentially explaining the reduced linguistic advantages observed in later-born children. In addition, while sibling interactions can enhance social communication skills, they may not provide the same level of quality or complexity of language input as that offered by adult caregivers. This study’s result challenges the traditional assumption that first-born children enjoy a linguistic advantage. Several factors may help explain the apparent contradiction between this study’s findings and previous literature. Later-born children may benefit from sibling interaction, gaining language skills through overhearing and social engagement. In addition, personality traits such as adaptability, along with evolving parenting styles and increased access to digital learning resources, may contribute to better speech outcomes.^[45–48]^

Other perinatal factors, including maternal illnesses during pregnancy, difficult delivery, prematurity, low birth weight, and postnatal health problems, showed an insignificant association with DLD. The lack of associations may also be partially explained by the sampling approach, which could have impacted the distribution of relevant risk factors in the study population. Future research should consider incorporating variables such as the home literacy environment and paternal mental health, as these may significantly influence children’s language outcomes. These factors can interact with other developmental risk elements in complex ways, and their inclusion would provide a more comprehensive understanding of language development trajectories.

### 4.1. Implications of the study

The study revealed a high prevalence of DLD, with more than one-third of the studied children aged 6 years or below in Abha, Saudi Arabia experiencing speech and language impairments. The findings underscore the importance of prioritizing DLD as a public health concern. In addition, it is essential to implement nationwide screening programs. These programs should target children at early stages in community settings, such as nurseries and schools to ensure timely identification and intervention for language delays. Maternal education campaigns should be developed to raise awareness about child developmental milestones, the importance of interactive communication, breastfeeding promotion, and strategies to promote language skills. In addition, policy initiatives that prioritize early childhood development should be strengthened, including increased funding for speech and language programs, training for specialists, and community awareness campaigns. Finally, to address DLD comprehensively, a multidisciplinary approach involving pediatricians, speech-language pathologists, educators, and psychologists is recommended. Moreover, culturally adapted intervention programs should be developed to meet the specific needs of Saudi children and families.

### 4.2. Limitations

Despite the novelty of the research topic, this study has several limitations. First, using non-random sampling techniques, namely, convenience and snowball techniques, may limit the generalizability of the findings to the broader Saudi population. The second is the inherited limitations of cross-sectional design in assessing the causality of speech delay and the studied predictors. Another limitation of a cross-sectional survey is the potential liability for recall bias. Future research is recommended to use a more representative sampling approach that includes diverse population groups across all regions of Saudi Arabia to ensure broader generalizability and more accurate identification of risk factors.

## 5. Conclusion

Nearly one-third of the studied participants in the Aseer region of Saudi Arabia had DLD, with significant associations observed between DLD and child age, maternal education levels, and feeding practices. Implementation of community-based screening and targeted intervention programs, particularly for at-risk groups, are mandatory to address this public health problem and mitigate its long-term effects on the Saudi community.

## Acknowledgments

The authors extend their appreciation to the Deanship of Research and Graduate Studies at King Khalid University for funding this work through the Small Research Project under grant RGP 1/188/45.

## Author contributions

**Conceptualization:** Saleh M. Al-Qahtani, Hoda Ali Ahmed Shiba, Hebatalla Abdelmaksoud Abdelmonsef Ahmed, Ayed A. Shati, Ramy Mohamed Ghazy.

**Funding acquisition:** Saleh M. Al-Qahtani.

**Methodology:** Saleh M. Al-Qahtani, Hoda Ali Ahmed Shiba, Hebatalla Abdelmaksoud Abdelmonsef Ahmed, Ramy Mohamed Ghazy.

**Supervision:** Saleh M. Al-Qahtani, Hebatalla Abdelmaksoud Abdelmonsef Ahmed, Ramy Mohamed Ghazy.

**Writing – original draft:** Saleh M. Al-Qahtani, Hoda Ali Ahmed Shiba, Hebatalla Abdelmaksoud Abdelmonsef Ahmed, Ayed A. Shati, Layan Saeed Alshmrani, Razan Mubarak Alqahtani, Reema Saeed Almater, Rawan Alqahtani, Reema Mohammed Abdullah Alshehri, Yara Ahmed Salem Alshehri, Lamia Ahmed M. Saddah, Ramy Mohamed Ghazy.

**Writing – review & editing:** Saleh M. Al-Qahtani, Hoda Ali Ahmed Shiba, Hebatalla Abdelmaksoud Abdelmonsef Ahmed, Ayed A. Shati, Ramy Mohamed Ghazy.

**Investigation:** Hoda Ali Ahmed Shiba, Hebatalla Abdelmaksoud Abdelmonsef Ahmed, Layan Saeed Alshmrani, Razan Mubarak Alqahtani, Reema Saeed Almater, Rawan Alqahtani, Reema Mohammed Abdullah Alshehri, Yara Ahmed Salem Alshehri, Lamia Ahmed M. Saddah, Ramy Mohamed Ghazy.

**Project administration:** Hoda Ali Ahmed Shiba, Ramy Mohamed Ghazy.

**Validation:** Hoda Ali Ahmed Shiba, Hebatalla Abdelmaksoud Abdelmonsef Ahmed.

**Data curation:** Hebatalla Abdelmaksoud Abdelmonsef Ahmed.

**Formal analysis:** Hebatalla Abdelmaksoud Abdelmonsef Ahmed.

**Software:** Hebatalla Abdelmaksoud Abdelmonsef Ahmed.

**Visualization:** Hebatalla Abdelmaksoud Abdelmonsef Ahmed.

## Supplementary Material

## References

[1] Baker L. Anatomy and physiology of the speech mechanism & features of the human body. Fishladder A Student J Art Writ. 2018;16:2–7.

[2] Liang WHK Gn LWE Tan YCD Tan GH. Speech and language delay in children: a practical framework for primary care physicians. Singapore Med J. 2023;64:745–50. 38047330 10.4103/singaporemedj.SMJ-2022-051 PMC10775292

[3] Dodd K Holm A Crosbie S. Treating inconsistent speech disorders. Differ Diagn Treat Child Speech Disord. 2013;10:182–201.

[4] McLaughlin MR. Speech and language delay in children. Am Fam Physician. 2011;83:1183–8. 21568252

[5] Aslam I Mumtaz N Saqulain G. Prevalence of speech sound disorders among primary school children. J Islam Med Dent Coll. 2020;9:195–200.

[6] Law J Dennis JA Charlton JJ. Speech and language therapy interventions for children with primary speech and/or language disorders. Cochrane Database Syst Rev. 2017;2017:CD012490. 10.1002/14651858.CD004110 PMC8407295 12918003

[7] Zubrick SR Taylor CL Rice ML Slegers DW. Late language emergence at 24 months: an epidemiological study of prevalence, predictors, and covariates. J Speech Lang Hear Res. 2007;50:1562–92. 18055773 10.1044/1092-4388(2007/106) PMC3521638

[8] Diepeveen FB Dusseldorp E Bol GW Oudesluys-Murphy AM Verkerk PH. Failure to meet language milestones at two years of age is predictive of specific language impairment. Acta Paediatr. 2016;105:304–10. 26585179 10.1111/apa.13271

[9] Soleimani F Bajalan Z. The relationship between growth indices at birth and developmental status in infants aged 6 to 18 months. Tehran Univ Med J. 2018;76:204–10.

[10] Bishop DVM Snowling MJ Thompson PA Greenhalgh T; CATALISE Consortium. CATALISE: a multinational and multidisciplinary delphi consensus study. Identifying language impairments in children. PLoS One. 2016;11:e0158753. 27392128 10.1371/journal.pone.0158753 PMC4938414

[11] Keshav M Julien L Miezel J. The role of technology in era 5.0 in the development of Arabic language in the world of education. JILTECH J Int Ling Technol. 2022;1:79–98.

[12] Ethnologue. What are the top 200 most spoken languages? 2025. https://www.ethnologue.com/insights/ethnologue200/. Accessed April 20, 2025.

[13] Rakhlin NV Aljughaiman A Grigorenko EL. Assessing language development in Arabic: the Arabic language: Evaluation of function (ALEF). Appl Neuropsychol Child. 2021;10:37–52. 31076015 10.1080/21622965.2019.1596113

[14] Alzahrani LD Aldharman SS Almuzaini AS. Prevalence and risk factors of speech delay in children less than seven years old in Saudi Arabia. Cureus. 2023;15:e48567. 38073978 10.7759/cureus.48567 PMC10710226

[15] Cronin P Reeve R Mccabe P Viney R Goodall S. The impact of childhood language difficulties on healthcare costs from 4 to 13 years: Australian longitudinal study. Int J Speech Lang Pathol. 2017;19:381–91. 27712125 10.1080/17549507.2016.1216599

[16] Ghazy RM Ali Ahmed Shiba H Alsaleem SA. The Stigma associated with speech delay in the Middle East and North Africa. In: Bennett G Goodall E, eds. The Palgrave Encyclopedia of Disability. Palgrave Macmillan, Cham, Switzerland: Springer Nature; 2025.

[17] Wallace IF Berkman ND Watson LR. Screening for speech and language delay in children 5 years old and younger: a systematic review. Pediatrics. 2015;136:e448–62. 26152671 10.1542/peds.2014-3889

[18] Kumar A Zubair M Gulraiz A. An assessment of risk factors of delayed speech and language in children: a cross-sectional study. Cureus. 2022;14:e29623. 36320964 10.7759/cureus.29623 PMC9608900

[19] Arshad H Ghayas MS Ghyas R Shabbir M. Patterns and risk factors associated with speech sounds and language disorders in Pakistan. Ann King Edward Med Univ. 2014;19:226.

[20] Torabi F Akbari SAA Amiri S Soleimani F Majd HA. Correlation between high-risk pregnancy and developmental delay in children aged 4-60 months. Libyan J Med. 2012;7. 10.3402/ljm.v7i0.18811 PMC3449296 23008747

[21] Dagvadorj A Ganbaatar D Balogun OO. Maternal socio-demographic and psychological predictors for risk of developmental delays among young children in Mongolia. BMC Pediatr. 2018;18:68. 29458342 10.1186/s12887-018-1017-y PMC5817794

[22] Bader B Coenen M Hummel J Schoenweger P Voss S Jung-Sievers C. Evaluation of community-based health promotion interventions in children and adolescents in high-income countries: a scoping review on strategies and methods used. BMC Public Health. 2023;23:845. 37165313 10.1186/s12889-023-15691-y PMC10170055

[23] Ehlen S Rehaag R. Analysis of comprehensive community-based health promotion approaches for children: health prospects in disadvantaged neighborhoods in Germany’s Ruhr area. Bundesgesundheitsblatt Gesundheitsforschung Gesundheitsschutz. 2018;61:1260–9. 30182137 10.1007/s00103-018-2809-9

[24] Alshaikhi SA Alamri AM Alzilai IY. Diabetes and prediabetes prevalence through a community-based screening initiative in Alqunfudah, Saudi Arabia. Future Sci OA. 2024;10:FSO946. 38817391 10.2144/fsoa-2023-0208 PMC11137795

[25] Farag Mohamed H Allam MM Hamdy NA Ghazy RM Emara RH. A community pharmacy-based intervention in the matrix of type 2 diabetes mellitus outcomes (CPBI-T2DM): a cluster randomized controlled trial. Clin Med Insights Endocrinol Diabetes. 2021;14:11795514211056308. 10.1177/11795514211056307 PMC8619747 34840503

[26] Mengesha EW Tesfaye TD Boltena MT. Effectiveness of community-based interventions for prevention and control of hypertension in sub-Saharan Africa: a systematic review. PLOS Glob Public Health. 2024;4:e0003459. 39012878 10.1371/journal.pgph.0003459 PMC11251591

[27] Wamba AA Takah NF Johnman C. The impact of interventions for the primary prevention of hypertension in sub-Saharan Africa: a systematic review and meta-analysis. PLoS One. 2019;14:e0219623. 31323041 10.1371/journal.pone.0219623 PMC6641142

[28] Abu Hasseba A Elsaady S EL-Shoubary A HN Standardization, translation, and modification of the Preschool Language Scale – 4. Dissertation submitted for partial fulfillment of a Doctoral degree in Phoniatrics. Egypt: Faculty of Medicine, Ain Shams University; 2011. http://srv4.eulc.edu.eg/eulc_v5/Libraries/Thesis/BrowseThesisPages.aspx?fn=PublicDrawThesis&BibID=11036121. Accessed February 7, 2025.

[29] Kotby MN Khairy A Barakah M Rifaie N El-Shobary A. Language testing of Arabic speaking children. Proceedings of the XXIII World Congress of the International Association of Logopedics and Phoniatrics. 1995.

[30] Ebert KD Ochoa-Lubinoff C Holmes MP. Screening school-age children for developmental language disorder in primary care. Int J Speech Lang Pathol. 2020;22:152–62. 31262202 10.1080/17549507.2019.1632931 PMC6938570

[31] Paradis J Emmerzael K Duncan TS. Assessment of English language learners: using parent report on first language development. J Commun Disord. 2010;43:474–97. 20304411 10.1016/j.jcomdis.2010.01.002

[32] Metwally AM Abdallah AM El-Din EMS. Screening and determinant of suspected developmental delays among Egyptian preschool-aged children: a cross-sectional national community-based study. BMC Pediatr. 2023;23:1–18. 37858055 10.1186/s12887-023-04335-0 PMC10585886

[33] Almohammadi A Alaslani K Alroqi H. Late talking in young children in Saudi Arabia: identifying key risk factors. First Lang. 2025;45:323–75.

[34] Falster K Hanly M Banks E. Maternal age and offspring developmental vulnerability at age five: a population-based cohort study of Australian children. PLoS Med. 2018;15:e1002558. 29689098 10.1371/journal.pmed.1002558 PMC5915778

[35] Lanjekar PD Joshi SH Lanjekar PD Wagh V. The effect of parenting and the parent-child relationship on a child’s cognitive development: a literature review. Cureus. 2022;14:e30574. 36420245 10.7759/cureus.30574 PMC9678477

[36] Chilosi AM Brovedani P Cipriani P Casalini C. Sex differences in early language delay and in developmental language disorder. J Neurosci Res. 2023;101:654–67. 34822733 10.1002/jnr.24976

[37] Määttä S Laakso ML Tolvanen A Ahonen T Aro T. Developmental trajectories of early communication skills. J Speech Lang Hear Res. 2012;55:1083–96. 22232414 10.1044/1092-4388(2011/10-0305)

[38] Sieverding M Hassan R. Associations between economic vulnerability and health and wellbeing in Egypt. Economic Research Forum (ERF); 2019.

[39] Metwally AM El-Sonbaty M Elmosalami D. Assessing the effective communication channels to reduce child and adolescent marriage in rural communities of Egypt. Open Access Maced J Med Sci. 2021;9:1288–99.

[40] Nomaguchi KM. Maternal employment, nonparental care, mother-child interactions, and child outcomes during preschool years. J Marriage Fam. 2006;68:1341–69.

[41] Iqbal M Rafique G Ali S. The effect of breastfeeding on the cognitive and language development of children under 3 years of age: results of “Balochistan-early childhood development project”. J Gen Pract. 2017;05.

[42] Abida LL Murti B Prasetya H. Meta-analysis: the effect of breast milk on child language. J Matern Child Health. 2021;5:579–89.

[43] Houmark MA. First Among Equals? How Birth Order Shapes Child Development. MPRA Paper. University Library of Munich. 2023;119325:1–62.

[44] Zajonc RB Markus GB. Birth order and intellectual development. Psychol Rev. 1975;82:74–88.

[45] Nafissi Z Vosoughi M. A critical meta-analytic exploration of birth order effect on L1 onset time of speaking and language development progression; is the pointer towards first or later Borns? Theory Pract Lang Stud. 2015;5:1960.

[46] Sulloway FJ. Birth order, sibling competition, and human behavior. Conceptual challenges in evolutionary psychology: Innovative research strategies. Springer; 2001.

[47] Oshima‐Takane Y Goodz E Derevensky JL. Birth order effects on early language development: do secondborn children learn from overheard speech? Child Dev. 1996;67:621–34.

[48] Zambrana IM Ystrom E Pons F. Impact of gender, maternal education, and birth order on the development of language comprehension: a longitudinal study from 18 to 36 months of age. J Dev Behav Pediatr. 2012;33:146–55. 22237556 10.1097/DBP.0b013e31823d4f83

