# Supplementary material for: Prevalence and risk factors of developmental language delay in a sample of children aged <6 years old in the Aseer region, Saudi Arabia: A community-based study
Source: Medicine (Baltimore). 2025 Jul 25;104(30):e43459. doi: 10.1097/MD.0000000000043459 (PMC12303488; doi:10.1097/MD.0000000000043459)
Supplement: Supplementary file 1 [file medi-104-e43459-s001.docx]

**Table S1. Post-hoc pairwise comparisons of PLS-4 standardized scores across different age groups**

| **Comparison of Age Groups** | | **Auditory Comprehension** | **Expressive Communication** | **Total Score** |
| --- | --- | --- | --- | --- |
| **0–12 months (Infants)** | 13–24 months (Toddlers) | 0.999 | 0.468 | 0.002* |
|  | 25–36 months (Early preschoolers) | 0.012* | 0.032* | <0.001* |
|  | 37–48 months (Preschoolers) | <0.001* | <0.001* | <0.001* |
|  | 49–60 months (Late preschoolers) | 0.005* | 0.018* | <0.001* |
|  | 61–72 months (Kindergarten Age) | <0.001* | 0.189 | <0.001* |
| **13–24 months (Toddlers)** | 25–36 months (Early preschoolers) | 0.479 | 0.999 | 0.999 |
|  | 37–48 months (Preschoolers) | 0.001* | 0.040* | 0.120 |
|  | 49–60 months (Late preschoolers) | 0.217 | 0.999 | 0.999 |
|  | 61–72 months (Kindergarten Age) | 0.002* | 0.999 | 0.894 |
| **25–36 months (Early preschoolers)** | 37–48 months (Preschoolers) | 0.520 | 0.129 | 0.999 |
|  | 49–60 months (Late preschoolers) | 0.999 | 0.999 | 0.999 |
|  | 61–72 months (Kindergarten age) | 0.429 | 0.999 | 0.999 |
| **37–48 months (Preschoolers)** | 49–60 months (Late preschoolers) | 0.999 | 0.521 | 0.999 |
|  | 61–72 months (Kindergarten age) | 0.999 | 0.287 | 0.999 |
| **49–60 months (Late preschoolers)** | 61–72 months (Kindergarten age) | 0.999 | 0.999 | 0.999 |

* Significant. Preschool Language Scale, Fourth Edition (PLS-4)
