# Supplementary material for: Prevalence and risk factors of developmental language delay in a sample of children aged <6 years old in the Aseer region, Saudi Arabia: A community-based study
Source: Medicine (Baltimore). 2025 Jul 25;104(30):e43459. doi: 10.1097/MD.0000000000043459 (PMC12303488; doi:10.1097/MD.0000000000043459)
Supplement: Supplementary file 2 [file medi-104-e43459-s002.docx]

**Table S2. Univariate logistic regression analysis of speech impairment according to child and family demographic characteristics**

| Studied variables | | Odds ratio (95%CI) | p-value | |
| --- | --- | --- | --- | --- |
| Gender  *(reference = Female)* | **Male** | 0.886(0.591:1.329) | 0.558 |  |
| Residence  *(reference= Urban)* | **Rural** | 0.831(0.482:1.431) | 0.504 |  |
| Mother's age at child's birth (years) | | 0.998(0.928:1.074) | 0.958 |  |
| Father’s age at child’s birth (years) | | 1.002(0.945:1.062) | 0.947 |  |
| Father's Education  *(reference= University)* | **Primary/Intermediate** | 2.027(0.759-5.417) | 0.159 |  |
|  | **Secondary** | 1.095(0.659-1.821) | 0.726 |  |
|  | **Postgraduate** | 0.491(0.193-1.252) | 0.136 |  |
| Mother's Education  *(reference=University)* | **Illiterate** | 1.127(0.202-6.278) | 0.892 |  |
|  | **Primary/Intermediate** | 1.448(0.602-3.486) | 0.408 |  |
|  | **Secondary** | 2.123(1.325-3.403) | 0.002* |  |
|  | **Postgraduate** | 1.202(0.490-2.950) | 0.688 |  |
| Father's Occupation  *(reference= Government Employee)* | **Private business** | 0.610(0.348-1.068) | 0.084 |  |
|  | **Unemployed** | 1.030(0.329-3.219) | 0.960 |  |
| Mother's Occupation  *(reference= Employed)* | **Unemployed** | 1.036(0.681-1.577) | 0.867 |  |
| Family Income  *(reference = 5000-15000 SAR)* | **< 5000- SAR** | 1.340(0.588-3.054) | 0.486 |  |
|  | **>15000- 20000SAR** | 0.827(0.517-1.322) | 0.426 |  |
|  | **> 20000 SAR** | 1.941(0.931-4.047) | 0.077 |  |
| Parental relation (Consanguinity) | | 0.718(0.445:1.157) | 0.174 |  |
| Number of children in family | | 1.173(0.932:1.476) | 0.174 |  |
| Child's birth order | | 0.453(0.263:0.780) | 0.004* |  |
| Family history of speech/language disorder | | 1.729(0.788:3.794) | 0.172 |  |
| How much screen time does the child have (TV, mobile phone, or laptop)?  *(reference=Less than 2 hours)* | **More than 2 hours** | 1.331(0.829:2.135) | 0.236 |  |

*Significant, SAR: Saudi Arabian Riyal (1USD = 3.75 SAR). CI : Confidence interval
