# Supplementary material for: Prevalence and risk factors of developmental language delay in a sample of children aged <6 years old in the Aseer region, Saudi Arabia: A community-based study
Source: Medicine (Baltimore). 2025 Jul 25;104(30):e43459. doi: 10.1097/MD.0000000000043459 (PMC12303488; doi:10.1097/MD.0000000000043459)
Supplement: Supplementary file 3 [file medi-104-e43459-s003.docx]

**Table S3. Univariate logistic regression analysis of speech impairment according to perinatal and postnatal health history**

| Studied variables | | Odds ratio (95%CI) | p-value |
| --- | --- | --- | --- |
| Did the mother suffer from any illnesses during pregnancy?  *(reference = No)* | **Yes** | 1.036(0.476:2.252) | 0.930 |
| Was the delivery difficult?  *(reference = No)* | **Yes** | 0.705(0.360:1.380) | 0.307 |
| Was the delivery premature (before 37 weeks of gestation)?  *(reference = No)* | **Yes** | 1.593(0.644:3.941) | 0.313 |
| Was the child's weight less than 2500 grams at birth?  *(reference = No)* | **Yes** | 0.599(0.266:1.350) | 0.216 |
| Did the child experience any health problems after birth?  *(reference= No)* | **Yes** | 0.713(0.396:1.283) | 0.259 |
| How was the child's nutrition?  *(reference = Bottle feeding)* | **Breastfeeding** | 0.404(0.262:0.625) | <0.001* |
| Use of pacifiers  *(reference = No)* | **Yes** | 1.03(0.674:1.574) | 0.891 |
| Is there any problem with hearing?  *(reference = No)* | **Yes** | 1.988(0.440:8.989) | 0.372 |
| Has the child ever had a middle ear infection?  *(reference = No)* | **Yes** | 0.72(0.341:1.520) | 0.389 |
| Has the child ever had any other ear, nose, or throat-related illnesses?  *(reference = No)* | **Yes** | 0.719(0.385:1.345) | 0.302 |
| Does the child have any deformity in the mouth or pharynx?  *(reference = No)* | **Yes** | 3.387(0.191:60.175) | 0.406 |

*Significant.CI: Confidence interval
